# Genetically predicted adiponectin causally reduces the risk of chronic kidney disease, a bilateral and multivariable mendelian randomization study

**DOI:** 10.3389/fgene.2022.920510

**Published:** 2022-07-26

**Authors:** Ruicheng Wu, Peiyi Luo, Min Luo, Xiaoyu Li, Xin Zhong, Qiang He, Jie Zhang, Yangchang Zhang, Yang Xiong, Ping Han

**Affiliations:** ^1^ Department of Urology, West China Hospital, Sichuan University, Chengdu, China; ^2^ Department of Nephrology, West China Hospital of Sichuan University, Chengdu, China; ^3^ Kidney Research Institute, West China Hospital of Sichuan University, Chengdu, China; ^4^ Laboratory of Innovation, Basic Medical Experimental Teaching Centre, Chongqing Medical University, Chongqing, China; ^5^ Department of Neurosurgery, West China Hospital, Sichuan University, Chengdu, China; ^6^ Department of Epidemiology and Health Statistics, School of Public Health and Management, Chongqing Medical University, Chongqing, China

**Keywords:** adiponectin, chronic kidney disease, estimated glomerular filtration rate, causal estimates, mendelian randomization

## Abstract

**Background:** It is not clarified whether the elevation of adiponectin is the results of kidney damage, or the cause of kidney function injury. To explore the causal association of adiponectin on estimated glomerular filtration rate (eGFR) and chronic kidney disease (CKD), this study was performed.

**Materials and methods:** The genetic association of adiponectin were retrieved from one genome-wide association studies with 39,883 participants. The summary-level statistics regarding the eGFR (133,413 participants) and CKD (12,385 CKD cases and 104,780 controls) were retrieved from the CKDGen consortium in the European ancestry. Single-variable Mendelian randomization (MR), bilateral and multivariable MR analyses were used to verify the causal association between adiponectin, eGFR, and CKD.

**Results:** Genetically predicted adiponectin reduces the risk of CKD (OR = 0.71, 95% CI = 0.57–0.89, *p* = 0.002) and increases the eGFR (*β* = 0.014, 95% CI = 0.001–0.026, *p* = 0.034) by the inverse variance weighting (IVW) estimator. These findings remain consistent in the sensitivity analyses. No heterogeneity and pleiotropy were detected in this study (*P* for MR-Egger 0.617, *P* for global test > 0.05, and *P* for Cochran’s Q statistics = 0.617). The bilateral MR identified no causal association of CKD on adiponectin (OR = 1.01, 95% CI = 0.96–1.07, *p* = 0.658), nor did it support the association of eGFR on adiponectin (OR = 0.86, 95% CI = 0.68–1.09, *p* = 0.207) by the IVW estimator. All the sensitivity analyses reported similar findings (*p* > 0.05). Additionally, after adjusting for cigarette consumption, alcohol consumption, body mass index, low density lipoprotein, and total cholesterol, the ORs for CKD are 0.70 (95% CI = 0.55–0.90, *p* = 0.005), 0.75 (95% CI = 0.58–0.97, *p* = 0.027), 0.82 (95% CI = 0.68–0.99, *p* = 0.039), 0.74 (95% CI = 0.59–0.93, *p* = 0.011), and 0.79 (95% CI = 0.61–0.95, *p* = 0.018), respectively.

**Conclusion**: Using genetic data, this study provides novel causal evidence that adiponectin can protect the kidney function and further reduce the risk of CKD.

## Introduction

Characterized by the chronic decrease in glomerular filtration rate due to various factors, chronic kidney disease (CKD) is receiving high levels of public attention. According to several large-scale epidemiological studies, the prevalence is 10.8% in Chinese adults (Zhang et al., 2012) and 6.9% in American adults aged 20 years or older (Murphy et al., 2016). Worse yet, a review by Glassock et al. (2017) showed that this common disorder significantly increases the risks of morbidity and mortality and heavily burdens the patients and health care system. Given the enormous high-risk population and persistent burden caused by irreversible CKD, prevention prior to the onset of CKD is necessary.

Previous surveys have identified multiple factors associated with CKD, including diabetes, homocysteine (Yang et al., 2022), hypertension (Kim et al., 2021) and tea intake (Zhang et al., 2022). Among those factors, the role of adiponectin in the onset and progression of CKD has attracted researchers’ attention. Adiponectin is a plasma protein that is mainly secreted by adipose tissue (Fang and Judd, 2018). Cumulating studies have revealed that adiponectin can increase insulin sensitivity, suppress inflammation, and counteract atherosclerosis. It has been widely accepted that insulin insensitivity, inflammation, and atherosclerosis are key factors triggering kidney damage (McMaster et al., 2015; Opazo-Ríos et al., 2020). Hence, these protective effects may function in preventing kidney damage and the development of CKD. However, protective effects in animal models and cell lines have not been replicated in clinical investigations. Adiponectin-knockout mice were shown to have increased albuminuria and fusion of podocyte foot processes, indicating kidney damage (Sharma et al., 2008). Notably, further adiponectin treatment of adiponectin-knockout mice normalizes albuminuria. Hence, the protective role of adiponectin in this model seems clear. However, in clinical investigations, as described by Lim et al. (2015), CKD patients had higher circulating adiponectin than their non-CKD counterparts, and after adjusting for confounders, participants with adiponectin levels in the 75th percentile had a 2.88-fold higher risk of CKD than those with levels in the 25 percentile. The specific role of adiponectin in the onset and progression of CKD still needs further exploration using novel methods and adequate sample sizes.

To clarify the role of adiponectin, we adopted Mendelian randomization (MR) to address the discrepancies in previous studies. It should be noted that all previous studies in clinics had observational designs, which cannot overcome the bias from confounders or clarify the causal direction. The reverse causality of CKD on adiponectin can justify the finding that adiponectin increases the risk of CKD in observational studies (Lim et al., 2015). To some extent, the cross-sectional studies only reveal an association between adiponectin and CKD. The causal direction still remains uncertain. Besides, the results in cross-sectional studies are opposite, which may be attributed to the limited sample size, cross-sectional design, and especially confounding factors. These defects cannot be overcome by the observational design. MR is a widely applied method that adopts single nucleotide polymorphisms (SNPs) as instrumental variables (IVs) to replace the exposure (i.e., adiponectin) and outcome (i.e., eGFR, and CKD). The IVs are randomly assorted when two gametes fuse to form the zygote. Consequently, biases from confounders, such as BMI, cigarettes, and alcohol, are avoided, and a clear causal direction is disclosed. In this study, we used datasets from previous genome-wide association studies (GWASs) to perform bilateral and multivariable MR (MVMR) to examine the causal associations between adiponectin level, eGFR, and CKD, which may clarify the obscure results of previous studies.

## Materials and methods

### Data sources of adiponectin, estimated glomerular filtration rate, chronic kidney disease and adjusting factors

The genetic association of adiponectin level with CKD was derived from one meta-analysis with 39,883 participants (29,347 European whites, and 10,536 non-European individuals) (Dastani et al., 2012). This meta-analysis combined 33 cohorts across the ethnicities (26 European whites and seven non-European cohorts, including African American and East Asians). The genetic association of adiponectin was retrieved from the European whites to avoid population architecture bias. Circulating adiponectin level was determined by radioimmunoassay or ELISA methods and then subjected to natural log transformation. The SNPs associated with natural log transformed adiponectin levels were adjusted for age, sex, and body mass index (BMI). The ages of the enrolled participants ranged from 10 to 95 years old.

The summary-level statistics regarding the eGFR and CKD were retrieved from the CKDGen consortium (Pattaro et al., 2016). This consortium combined 49 predominantly population-based GWASs in populations of European descent using a meta-technique. Finally, a total of 117,165 participants (12,385 CKD cases and 104,780 controls) were enrolled to identify the SNPs significantly associated with CKD. In addition, 133,413 participants were genotyped to identify the SNPs significantly associated with eGFR. The calculation of eGFR was based on serum creatinine using the Modification of Diet in Renal Disease (MDRD) Study equation (Köttgen et al., 2009): eGFRcrea = 186.3 × serum creatinine (mg dl^−1^)− 1.154 × age − 0.203 × 0.742 (if female). Serum creatinine was measured by the enzymatic photometric assay, spectrophotometry, and modified kinetic Jaffe reaction in different cohorts (Pattaro et al., 2016). To diagnose CKD, the cutoff value of eGFR was set as < 60 ml/min/1.73 m^2^, as in previous studies (Xiong et al., 2021).

The genetic estimates of adjusting factors, including alcohol consumption, cigarette consumption, BMI, low-density lipoprotein (LDL), and total cholesterol (TC), were derived from previous cohorts (Willer et al., 2013; Yengo et al., 2018; Liu et al., 2019). Further detailed information with regard to the cohort information, samples, genotyping, and quality control criteria can be accessed in the original study.

### Genetic instrument selection

To retrieve the conditionally independent instrument variables (IVs), the statistical significance threshold of IVs was set as *p* < 5 × 10^−8^ with linkage disequilibrium (LD) *r* ^2^ < 0.001 at a window size of 10,000 Kb. The *p* value was determined based on the association between the SNPs and the phenotype (i.e., circulating adiponectin levels). Two palindromic SNPs (rs2980879 and rs7964945) were removed, and no proxy SNP was used in this study. Furthermore, to examine the reliability of the filtered SNPs, we used the MR-Steiger filtering approach to verify the causal direction of IVs. Insignificant results indicated that the variance explained in the exposure was lower than that in the outcome; thus, IVs with insignificant results should be removed from the analysis. In this step, no filtered SNP was pruned. Additionally, we also adopted the radial MR and MR pleiotropy residual sum and outlier (MR-PRESSO) methods to detect the outlier SNP, which may be responsible for the possible pleiotropy (Verbanck et al., 2018). No outliers were identified in this study. Finally, 10 SNPs were qualified and available in the outcome dataset and used as IVs to explore the causal association between adiponectin level, eGFR and CKD. Detailed information on the IVs is displayed in Supplementary Table 1. According to the calculation in the original study (Dastani et al., 2012), these SNPs explained 5% of the variance of natural log-transformed adiponectin levels.

### Main statistical analyses

The inverse variance weighting (IVW) method was performed to examine the causal estimates of genetically predicted adiponectin level on eGFR and CKD. First, we extracted and pruned the SNPs of adiponectin as described in *Genetic instruments selection*. Second, the remained SNPs, namely IVs in this study, were obtained from the outcome datasets and then harmonized for further regression analyses. In this step, the palindromic IVs were removed if existed. Finally, using the IVW method, the coefficients of IVs were combined as the final causal effect estimates. When heterogeneity existed, the random effect IVW (IVW-re) was seen as the main analysis, and when no heterogeneity was identified, the fixed effect IVW (IVW-fe) was deemed as the main analysis. The IVW method can yield consistent and unbiased causal effect estimates when assuming that all IVs are valid. All MR analyses were performed and all figures were generated by R 3.6.5 (R Foundation for Statistical Computing, Vienna, Austria), using the “TwoSampleMR”, “RadialMR”, “mr.raps”, and “forestplot” packages. *p* < 0.05 (two-sided) was set as the significance threshold.

### Sensitivity analyses

To further verify the conformity of the findings and detect the possible pleiotropy and heterogeneity, six other methods, including MR–Egger, weighted median, maximum likelihood, simple median, MR robust adjusted profile score (MR- RAPS), and MR-PRESSO were used. These approaches can yield unbiased causal effect estimates under different scenarios (i.e., invalid IVs, weak IVs, and pleiotropic IVs).

The MR–Egger approach (Burgess et al., 2019) can be applied to detect causal associations when all IVs are unqualified. In addition, the intercept term of the MR–Egger regression function can be used to quantify horizontal pleiotropy. Additionally, we also employed median-based methods (Bowden et al., 2016) (weighted median and simple median) to reanalyze the data, and these methods can detect causal effects when up to 50% IVs are invalid and have higher precision than the MR–Egger method. The maximum likelihood method (Milligan, 2003) has low standard error but may be biased by limited sample size. The MR-RAPS approach (Zhao et al., 2019) is statistically sound in the presence of weak IVs and remains robust when systematic and idiosyncratic pleiotropy exists. The MR-PRESSO method (Verbanck et al., 2018) can provide credible results when horizontal pleiotropy exists in less than 50% of IVs. Moreover, the global test of the MR-PRESSO method can be used to detect horizontal pleiotropy.

We also used three approaches to detect heterogeneity, including the IVW, ME-Egger, and maximum likelihood methods. The insignificant results of Cochran’s Q statistic indicate the absence of heterogeneity in all the MR analyses in this study. In addition, leave-one-out analysis was used, which excluded IVs one at a time and reanalyzed the data to verify the robustness of the conclusion.

### Reverse Mendelian randomization of the effect of genetically predicted estimated glomerular filtration rate and chronic kidney disease on adiponectin level

To test whether genetically predicted eGFR and CKD can causally affect the levels of circulating adiponectin, we extracted the IVs for eGFR and CKD and then performed two-sample MR. The lists of IVs used in the reverse MR analysis for CKD and eGFR were showed in Supplementary Tables 2, 3, respectively. All the parameters and statistical approaches were set as described before.

### Multivariable mendelian randomization of the effect of adiponectin level on chronic kidney disease adjusting for confounders

To obtain the direct causal effect of genetically predicted adiponectin level on CKD, we further adopted the MVMR framework to adjust for alcohol consumption, cigarette consumption, BMI, LDL, and TC. MVMR can be performed using a set of genetic variants, which predicts a set of exposure variables concurrently. The overlapping SNPs between adiponectin and the confounders were selected as the IVs and were then subjected to further IVW analysis. Based in this theory, MVMR is helpful in obtaining causal effect estimates of two or more exposures on an outcome and may help to understand whether both exposures exert a causal effect on the outcome.

## Results

### Causal effect estimates of adiponectin level on chronic kidney disease in single-variable mendelian randomization

**FIGURE 1:**
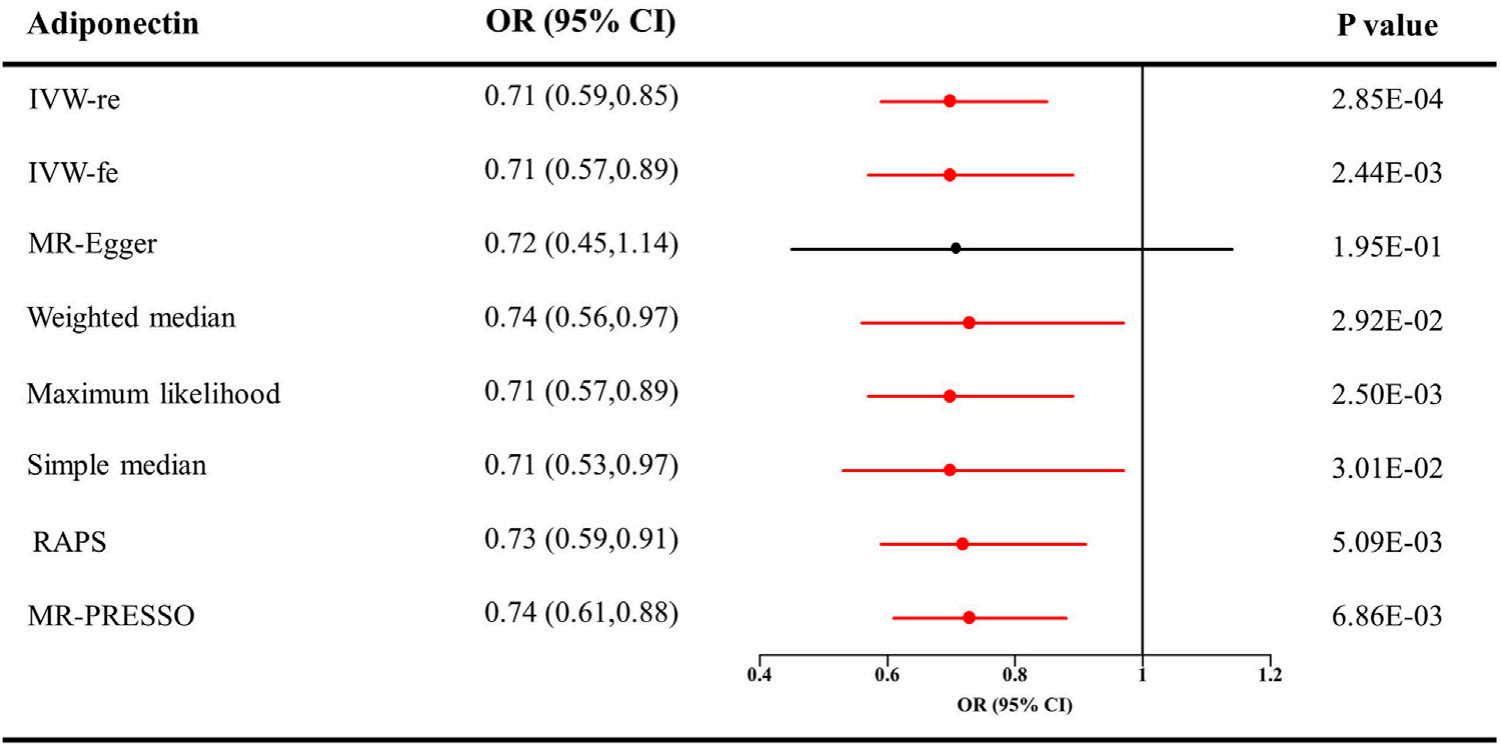
Causal estimates of adiponectin on CKD in single-variable MR. Red line, significant; Black line, not significant; OR, odds ratio; CI, confidence interval; IVW, Inverse variance weighted method; RAPS, robust adjusted profile score; MR, Mendelian randomization; PRESSO, pleiotropy residual sum and outlier; CKD, chronic kidney disease.

**FIGURE 2:**
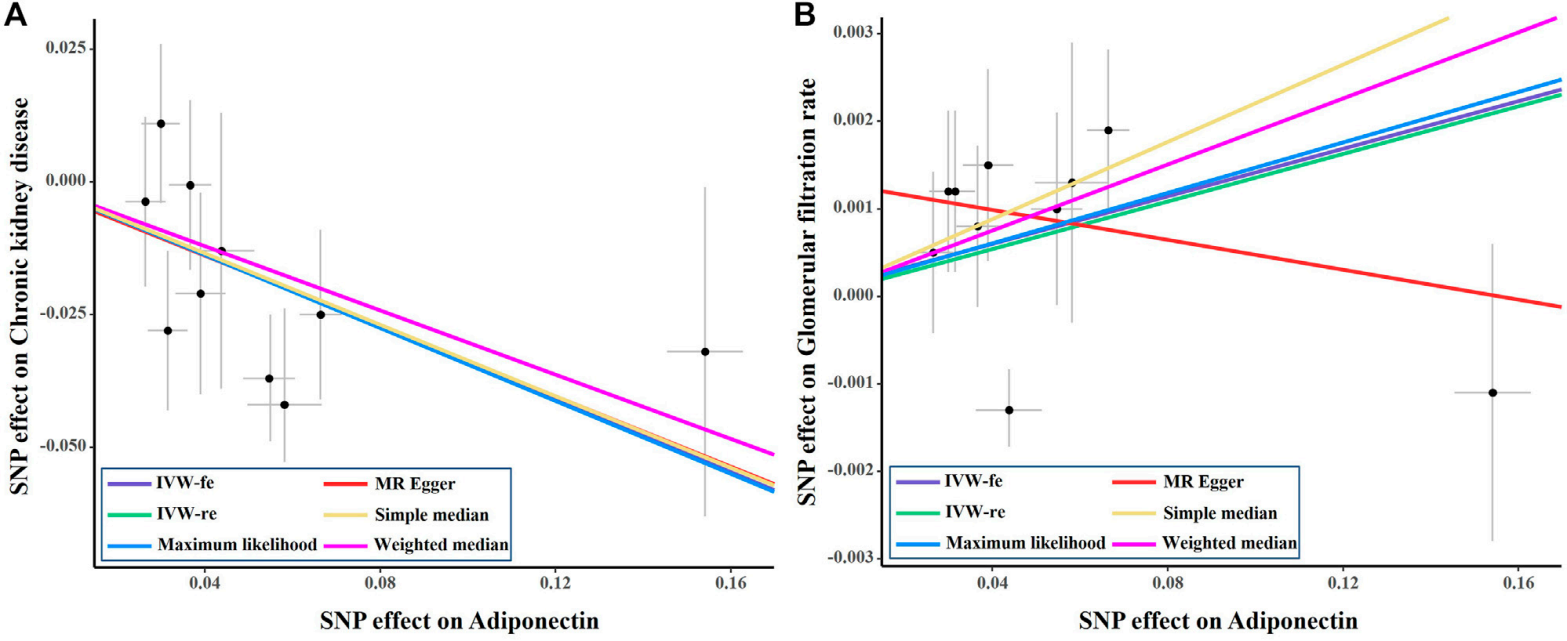
Scatter plot of the effect size of each SNP on adiponectin, eGFR and CKD in SVMR. SNP, single nucleotide polymorphism; eGFR, estimated glomerular filtration rate; IVW, Inverse variance weighted method; CKD, chronic kidney disease; MR, Mendelian randomization.

The causal effect estimates of adiponectin level on CKD are displayed in Figure 1. The IVW estimator reveals an OR of 0.71 (95% CI = 0.59–0.85, *p* = 0.0003 for the IVW-re estimator; 95% CI = 0.57–0.89, *p* = 0.002 for the IVW-fe estimator). The ORs for the MR–Egger, weighted median, maximum likelihood, simple median, MR-RAPS, and MR-PRESSO methods were 0.72 (95% CI = 0.45–1.14, *p* = 0.195), 0.74 (95% CI = 0.56–0.97, *p* = 0.03), 0.71 (95% CI = 0.57–0.89, *p* = 0.0002), 0.74 (95% CI = 0.53–0.97, *p* = 0.03), 0.73 (95% CI = 0.59–0.91, *p* = 0.005), and 0.74 (95% CI = 0.61–0.88, *p* = 0.007), respectively. For the sensitivity analyses, the results yielded by the MR–Egger method were insignificant, suggesting limited effectiveness. As shown in the scatter plot (Figure 2A), the SNP effect on CKD decreases with the increase in the SNP effect on adiponectin level, indicating that the circulating adiponectin decreases the risk of CKD.

In Table 1, Cochran’s Q statistics identified no signs of heterogeneity (Q statistics = 6.27 for the MR–Egger method, *p* = 0.617; 6.28 for the IVW estimator, *p* = 0.712; and 6.21 for the maximum likelihood approach, *p* = 0.719). No pleiotropy was detected by the ME-Egger estimator (intercept = −0.0006, *p* = 0.961) or the global test of the MR-PRESSO estimator (*p* > 0.05). The results of the leave-one-out analysis remained consistent when excluding one SNP at a time (Supplementary Figure 1A). The estimates of each IV are shown in Supplementary Figure 2A. The funnel plot displaying the heterogeneity is provided in Supplementary Figure 3A.

**TABLE 1:** MR estimates from each method of the causal effect of adiponectin on kidney function.

Traits	MR method	Cochran’s Q statistic	Heterogeneity *p* value	MR-Egger intercept	Intercept *p* value
CKD	MR-Egger	6.27	0.617	−0.0006	0.961
	IVW	6.28	0.712	—	—
	Maximum likelihood method	6.21	0.719	—	—
eGFR	MR-Egger	5.06	0.751	0.0013	0.089
	IVW	8.81	0.455	—	—
	Maximum likelihood method	8.77	0.459	—	—

MR: mendelian randomization; IVW: inverse variance weighted method.

### Causal effect estimates of adiponectin level on estimated glomerular filtration rate in single-variable mendelian randomization

**FIGURE 3:**
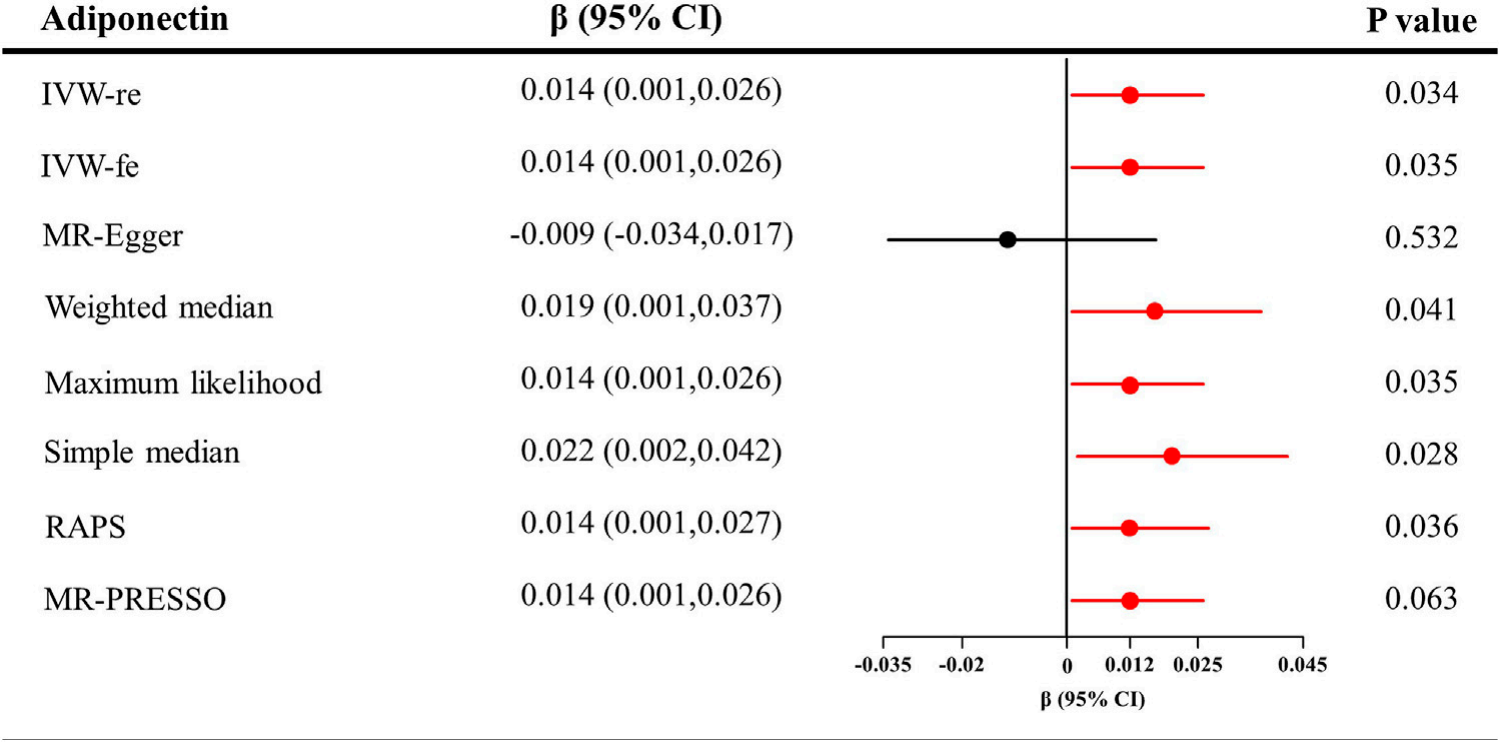
Causal estimates of adiponectin on eGFR in single-variable MR. Red line, significant; Black line, not significant; CI, confidence interval; eGFR, estimated glomerular filtration rate; IVW, Inverse variance weighted method; RAPS, robust adjusted profile score; MR, Mendelian randomization; PRESSO, pleiotropy residual sum and outlier.

As shown in Figure 3, genetically predicted higher circulating adiponectin level is causally associated with higher eGFR, suggesting a protective effect of adiponectin on eGFR decline. The *β* value was 0.014 (95% CI = 0.001–0.026, *p* = 0.034) by the IVW estimator. This finding remains consistent as evaluated by the weighted median (*β* = 0.019, 95% CI = 0.001–0.037, *p* = 0.041), maximum likelihood (*β* = 0.014, 95% CI = 0.001–0.026, *p* = 0.035), simple median (*β* = 0.022, 95% CI = 0.002–0.042, *p* = 0.028), MR-RAPS (*β* = 0.014, 95% CI = 0.001–0.027, *p* = 0.036), and MR-PRESSO (*β* = 0.014, 95% CI = 0.001–0.026, *p* = 0.063) methods. However, the MR–Egger estimator reveals an insignificant result (*β* = −0.009, 95% CI = −0.034–0.017, *p* = 0.532), suggesting limited effectiveness. The scatter plot visualizing the effect of adiponectin level on eGFR is displayed in Figure 2B.

In Table 1, the Cochran’s Q statistics were 5.06 for the MR–Egger method (*p* = 0.751), 8.81 for the IVW method (*p* = 0.455), and 8.77 for the maximum likelihood method (*p* = 0.459), indicating the absence of heterogeneity. Additionally, the intercept of MR–Egger regression was 0.0013 (*p* = 0.089), which is in line with the insignificant results of the global test in the MR-PRESSO estimator (*p* > 0.05). These analyses detected no signs of directional pleiotropy. The results of the leave-one-out analysis are displayed in Supplementary Figure 1B. The estimates of each IV are shown in Supplementary Figure 2B The funnel plot visualizing the heterogeneity is provided in Supplementary Figure 3B.

### Reverse Mendelian randomization between genetically predicted estimated glomerular filtration rate, chronic kidney disease and adiponectin level

**FIGURE 4:**
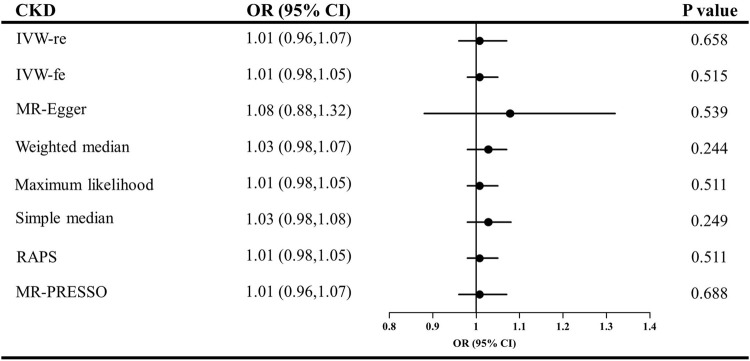
Causal estimates of CKD on adiponectin in single-variable MR. OR, odds ratio; CI, confidence interval; IVW, Inverse variance weighted method; RAPS, robust adjusted profile score; MR, Mendelian randomization; PRESSO, pleiotropy residual sum and outlier; CKD, chronic kidney disease.

**FIGURE 5:**
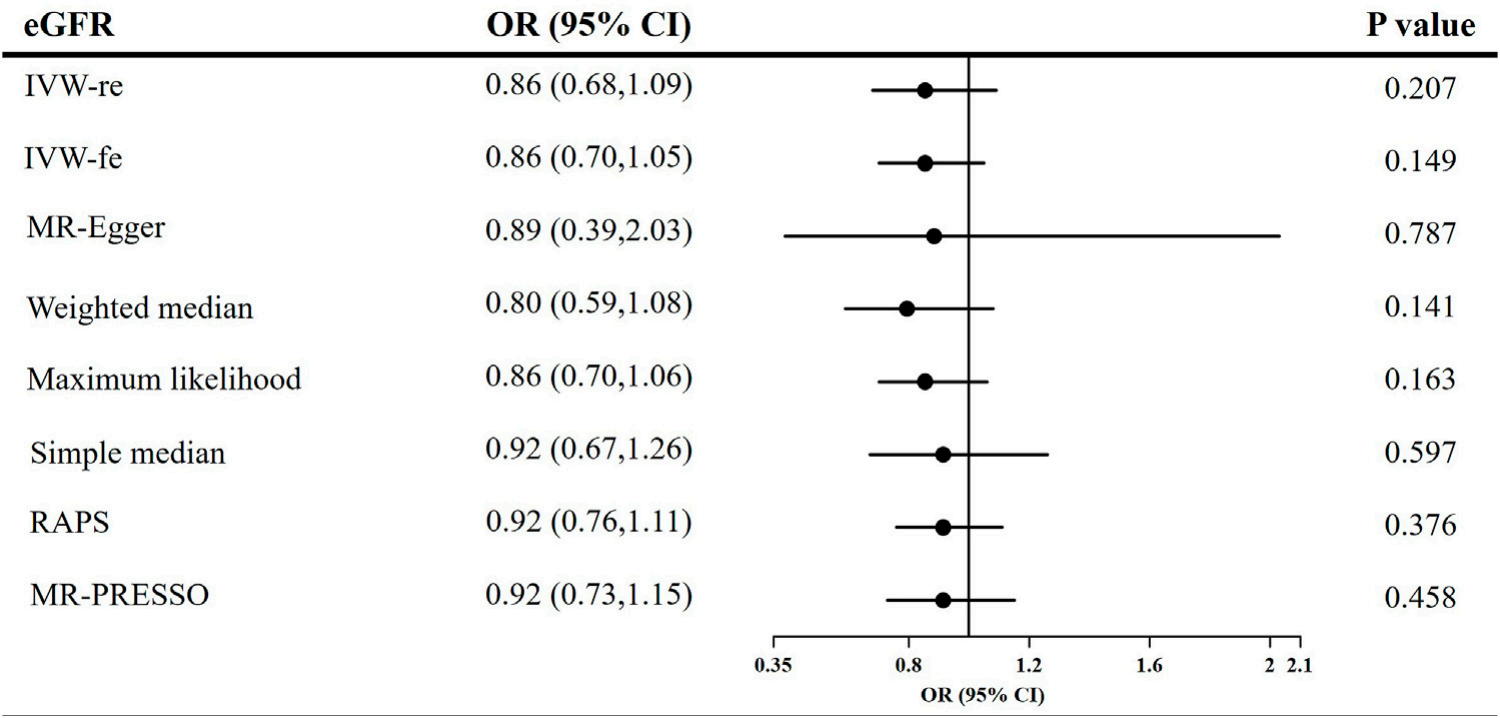
Causal estimates of eGFR on adiponectin in single-variable MR. OR, odds ratio; CI, confidence interval; eGFR, estimated glomerular filtration rate; IVW, Inverse variance weighted method; RAPS, robust adjusted profile score; MR, Mendelian randomization; PRESSO, pleiotropy residual sum and outlier.

The causal effect estimates of CKD and eGFR on circulating adiponectin level are shown in Figures 4, 5, respectively. In Figure 4, none of the analyses identified a causal association of CKD on adiponectin level. The ORs were 1.01 for the IVW (95% CI = 0.96–1.07, *p* = 0.658), maximum likelihood (95% CI = 0.98–1.05, *p* = 0.511), MR-RAPS (95% CI = 0.98–1.05, *p* = 0.511), and MR-PRESSO estimators (95% CI = 0.96–1.07, *p* = 0.688), 1.08 for the MR–Egger method (*p* = 0.539), and 1.03 for the weighted median and simple median estimators (*p* = 0.249). Additionally, in Figure 5, the results also do not support a causal association of eGFR on adiponectin level. The ORs were 0.86 for the IVW (95% CI = 0.68–1.09, *p* = 0.207) and maximum likelihood estimators (95% CI = 0.70–1.06, *p* = 0.163), 0.89 for the MR–Egger method (*p* = 0.787), 0.80 for the weighted median method (*p* = 0.141), and 0.92 for the simple median (95% CI = 0.67–1.26, *p* = 0.597), MR-RAPS (95% CI = 0.76–1.11, *p* = 0.376), and MR-PRESSO estimators (95% CI = 0.73–1.15, *p* = 0.458).

### Causal effect estimates of the effect of adiponectin level on chronic kidney disease in multivariable mendelian randomization

**FIGURE 6:**
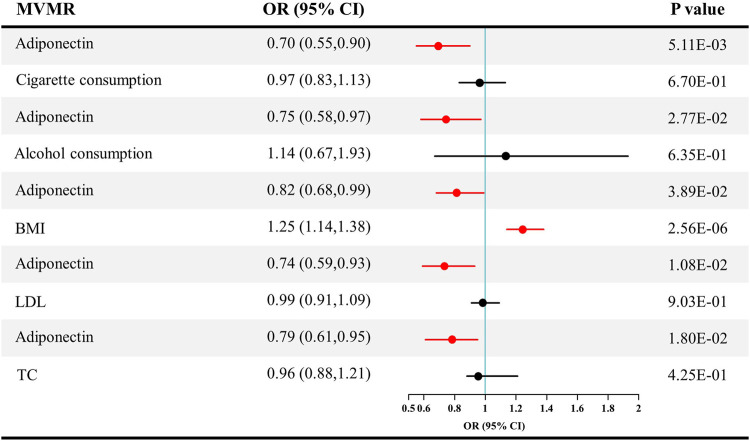
Causal estimates of adiponectin on CKD in multivariable MR. Red line, significant; Black line, not significant; OR, odds ratio; CI, confidence interval; LDL, low-density lipoprotein; TC, total cholesterol; CKD, chronic kidney disease; MVMR, multivariable Mendelian randomization.

After adjusting for confounders, the direct causal effect estimates of adiponectin level on CKD are provided in Figure 6. The ORs for CKD were 0.70 after adjusting for cigarette consumption (95% CI = 0.55–0.90, *p* = 0.005), 0.75 after adjusting for alcohol consumption (95% CI = 0.58–0.97, *p* = 0.027), 0.82 after adjusting for BMI (95% CI = 0.68–0.99, *p* = 0.039), 0.74 after adjusting for LDL (95% CI = 0.59–0.93, *p* = 0.011), and 0.79 after adjusting for TC (95% CI = 0.61–0.95, *p* = 0.018).

## Discussion

Using genetic data, this study identifies a causal association of adiponectin level on eGFR and CKD, instead of the reverse association. The effect sizes were similar across several sensitivity analyses, indicating a protective role of adiponectin on kidney function. This finding overcomes the biases from confounders and reverse causality, which clarifies the discrepancy in clinical surveys and animal models.

In contrast to our findings, the majority of clinical investigations support a role of adiponectin in the onset and progression of CKD. In a case–control study (Sedighi and Abediankenari, 2014) enrolling 42 CKD patients and 42 healthy controls, adiponectin in CKD patients was significantly higher than that in the healthy controls and was inversely correlated with eGFR. In another case–control study (Norata et al., 2010) with 360 participants, plasma adiponectin levels were found to be correlated with creatinine level and inversely correlated with eGFR. These findings support the hypothesis that adiponectin may lead to the deterioration of renal function. However, impaired kidney function may reduce the elimination of adiponectin and boost the secretion of adiponectin by adipose tissue, justifying those findings (Zha et al., 2017). The observed elevation of adiponectin may be the result of kidney damage rather than the cause of the kidney function injury. Given the difficulties and lack of rigorously designed randomized controlled trials, clarifying the causal direction and yielding unbiased conclusions from the results of previous clinical studies is difficult; thus, causal direction and bias were addressed in this study using MR analyses.

Some studies in clinical investigations and animal models may support our conclusion. In Japan, a prospective observational study (Kobayashi et al., 2018) enrolled 216 healthy volunteers with eGFR ≥ 60 ml/min/1.73 m^2^. After 5 years, the levels of plasma adiponectin remained similar in the kidney damage participants and the normal kidney function subjects. This finding does not support the role of adiponectin in the onset and progression of CKD but requires further verification due to the limited sample size of that study. Additionally, animal models that are not exposed to confounders, such as obesity, have also reported similar results. In adiponectin-knockout mice (Sharma et al., 2008), the lack of adiponectin can lead to an increase in albuminuria. Supplementation with adiponectin can reverse albuminuria to normal levels. Similarly, as revealed by Pereira et al. (2020), adiponectin-knockout mice show worse albuminuria than wild-type mice when exposed to a high-fat diet for 16 weeks. These animal studies observed a protective effect of adiponectin in CKD, in line with our findings under the MR framework. Therefore, it is possible that biases in observational studies lead to an opposite conclusion. Currently, after avoiding confounding factors, the true causal association of adiponectin level with eGFR and CKD is disclosed.

Several molecular mechanisms may explain the protective role of adiponectin in the onset and progression of CKD. First, adiponectin can improve insulin sensitivity and maintain homeostasis (Lin et al., 2013; Ahlstrom et al., 2017). Glucose dysregulation can trigger tubulointerstitial damage, such as that caused by macrophage infiltration and fibrosis, consequently leading to CKD (Cai et al., 2020). In addition, reduction of oxidative stress may be another mechanism of the protective role of adiponectin in CKD (Zhou et al., 2019). It was reported by Li et al. (2017) that adiponectin can lower reactive oxygen species (ROS) levels and increase superoxide dismutase (SOD) activity levels in an ischemia reperfusion mouse model, indicating the effects of antioxidant stress. Oxidative stress is an important activator of podocyte injury and proteinuria through the Wnt/β-catenin pathway (Zhou et al., 2019). Therefore, adiponectin may protect kidney function through its effect of antioxidant stress. These clear molecular mechanisms further clarify the causal association of adiponectin with eGFR and CKD, supporting our findings from prior studies.

There are still some merits and limitations of our study. The principal merit is the MR design of our study, which can avoid confounders and yield causal inferences. Under this framework, the different conclusions between animal models and clinical investigations are eliminated. However, a flaw of all two-sample MR studies is that the summary-level data cannot be used to explore the nonlinear relationship between adiponectin level, eGFR, and CKD. In addition, the genetic association in different ancestries may vary. Under different ethnic backgrounds, these findings need further verification.

In conclusion, this study provides novel causal evidence that adiponectin can protect kidney function and further reduce the risk of CKD. The clinical implications of the research findings mainly locate on the clear causal association between adiponectin and CKD. This indicates that intervening adiponectin may be beneficial to avoid or delay the onset and progression of CKD. Besides, for CKD patients with high adiponectin, reducing the concentration of adiponectin seems not necessary.

## Data Availability

The datasets presented in this study can be found in online repositories. The names of the repository/repositories and accession number(s) can be found in the article/Supplementary Material.

## Supplementary material

Supplementary Figure 1 The leave-one-out sensitivity analyses of adiponectin on CKD and eGFR. **(A)** The leave-one-out sensitivity analyses of adiponectin on CKD; **(B)** The leave-one-out sensitivity analyses of adiponectin on eGFR. SNP, single nucleotide polymorphism; eGFR, estimated glomerular filtration rate; CKD, chronic kidney disease; MR, Mendelian randomization.

Supplementary Figure 2 The results of MR analyses of causal associations between each adiponectin SNP on CKD and eGFR. **(A)** The forest plot of each adiponectin SNP on CKD; **(B)** The forest plot of each adiponectin SNP on eGFR. SNP, single nucleotide polymorphism; IVW, Inverse variance weighted method; eGFR, estimated glomerular filtration rate; CKD, chronic kidney disease; MR, Mendelian randomization.

Supplementary Figure 3 The funnel plot in SVMR. **(A)** The funnel plot of adiponectin on CKD; **(B)** The funnel plot of adiponectin on eGFR. IVW, Inverse variance weighted method; SVMR, single-variable Mendelian randomization. IV, instrumental variable; SE, standard error; MR, Mendelian randomization.

Supplementary Table 1 SNPs used as genetic instruments in the Mendelian randomization analyses. SNPs, single nucleotide polymorphisms.
