# Supplementary figures and images for: Genetically predicted adiponectin causally reduces the risk of chronic kidney disease, a bilateral and multivariable mendelian randomization study
Source: Front Genet. 2022 Jul 26;13:920510. doi: 10.3389/fgene.2022.920510 (PMC9360570; doi:10.3389/fgene.2022.920510)

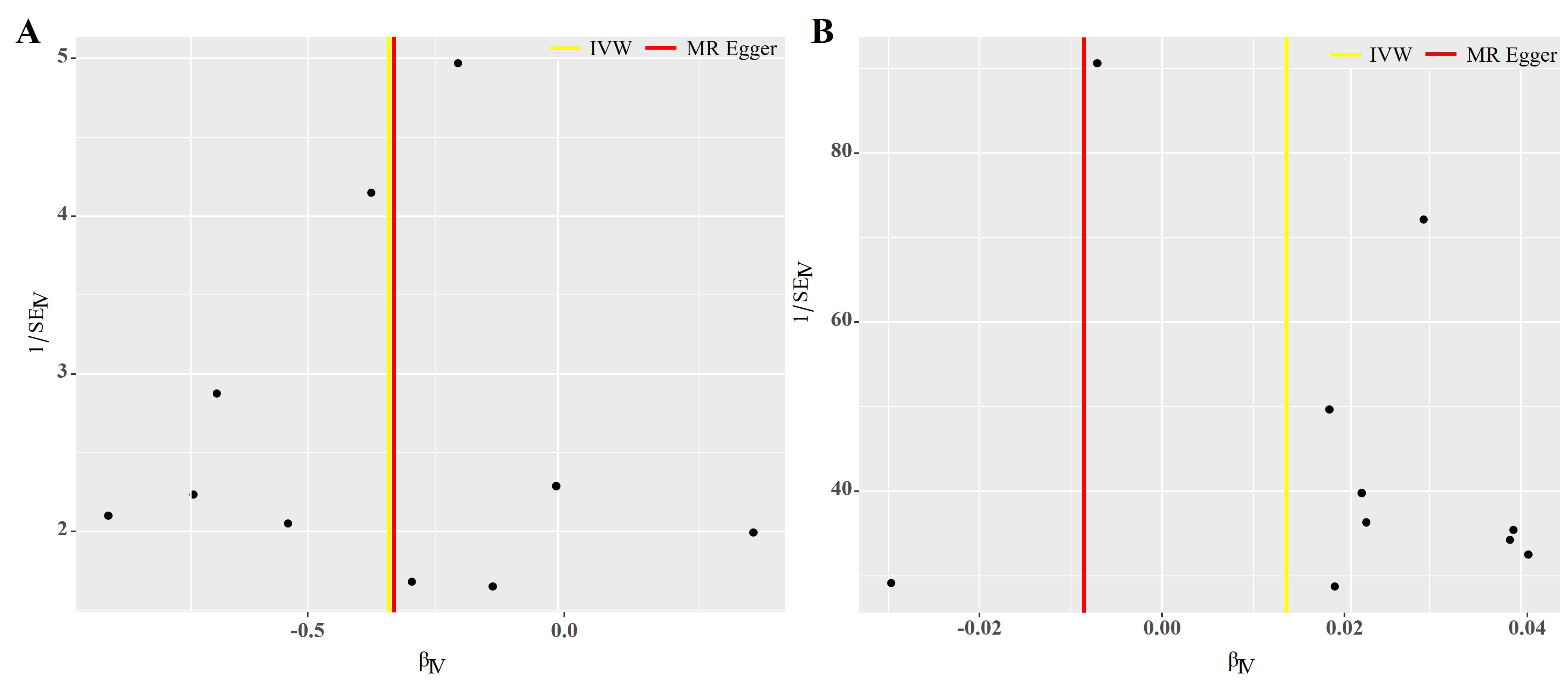

Supplement: Supplementary file 1 [file Image3.JPEG]

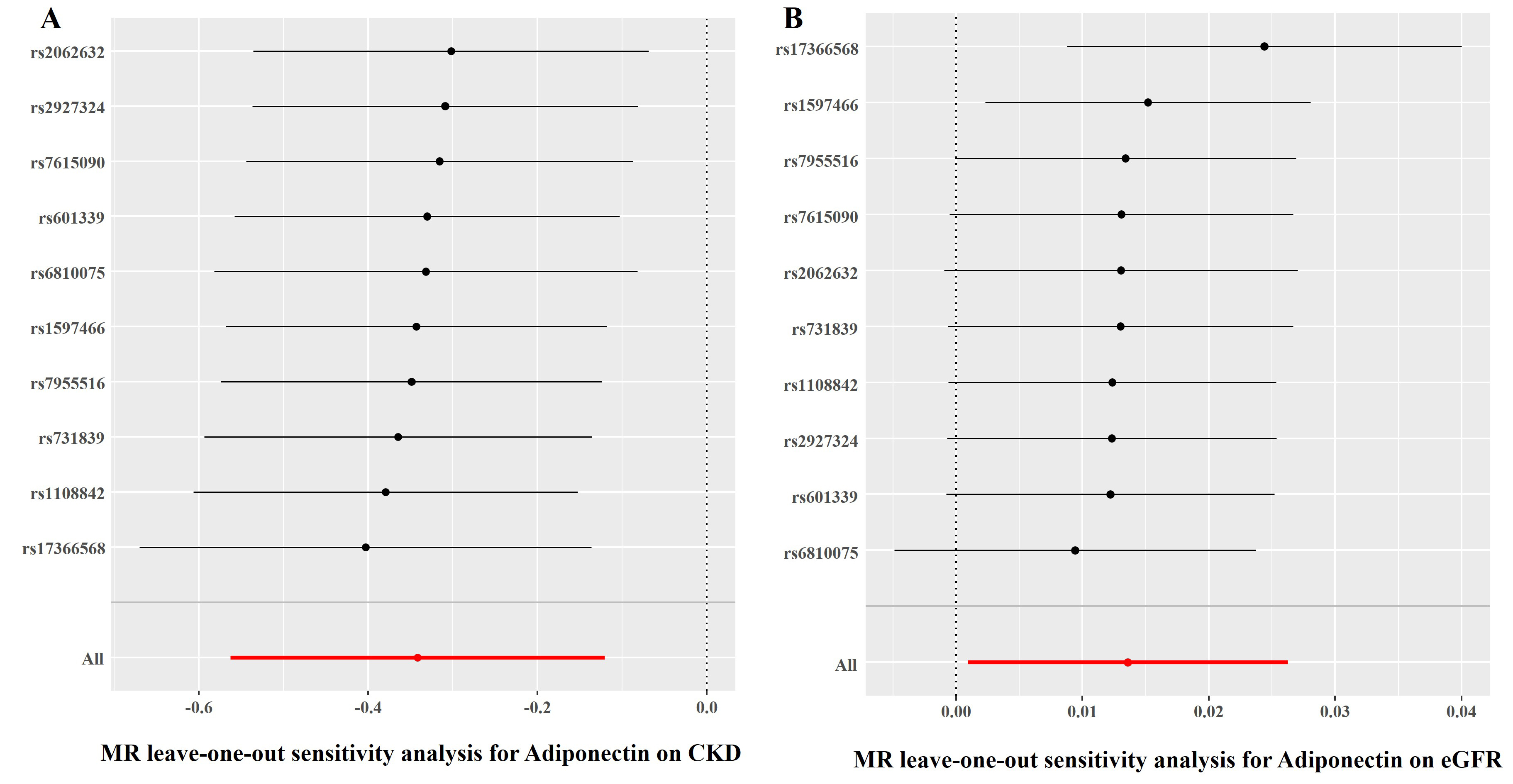

Supplement: Supplementary file 3 [file Image1.JPEG]

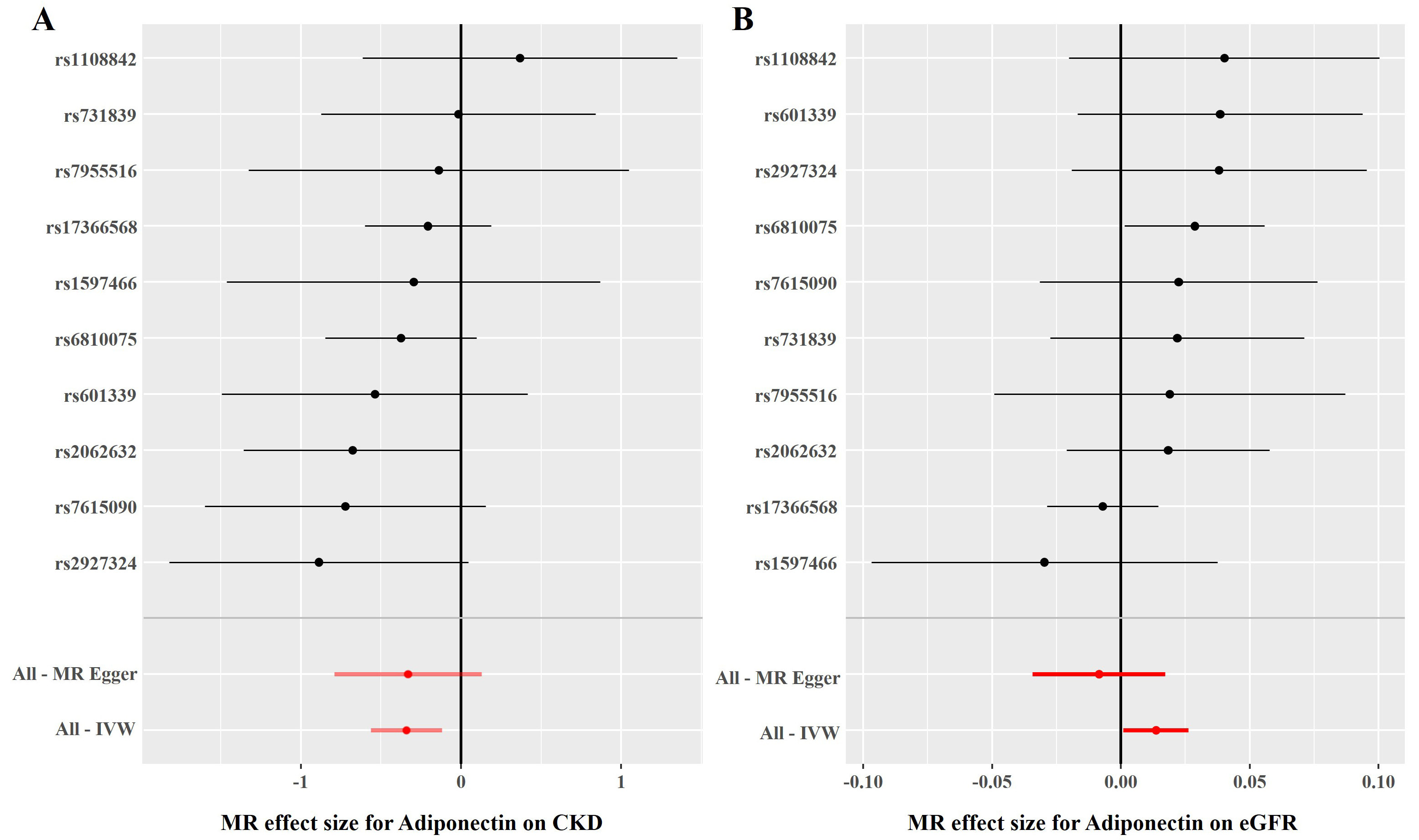

Supplement: Supplementary file 4 [file Image2.JPEG]
