# Supplementary material for: Genetically predicted adiponectin causally reduces the risk of chronic kidney disease, a bilateral and multivariable mendelian randomization study
Source: Front Genet. 2022 Jul 26;13:920510. doi: 10.3389/fgene.2022.920510 (PMC9360570; doi:10.3389/fgene.2022.920510)
Supplement: Supplementary file 2 [file Table1.DOCX]

| **SNP** | **chr** | **position** | **A1** | **A2** | **EAF** | **Beta** | **SE** | **P value** | **palindromic** | **Steiger** | **Steiger**  **P value** | **Nearby Gene** |
| --- | --- | --- | --- | --- | --- | --- | --- | --- | --- | --- | --- | --- |
| rs1108842 | 3 | 52720080 | C | A | 0.4583 | 0.029927 | 0.004382 | 3.66E-11 | FALSE | TRUE | 7.57E-09 | GNL3 |
| rs1597466 | 3 | 150055561 | T | G | 0.09167 | -0.043765 | 0.007542 | 1.89E-08 | FALSE | TRUE | 6.87E-07 | SIAH2 |
| rs17366568 | 3 | 186570453 | A | G | 0.90833 | -0.154104 | 0.008685 | 1.00E-200 | FALSE | TRUE | 3.33E-55 | ADIPOQ |
| rs2062632 | 3 | 186461181 | C | T | 0.6864 | -0.054666 | 0.005893 | 2.52E-19 | FALSE | TRUE | 1.06E-13 | KNG1 |
| rs2927324 | 16 | 81512821 | T | C | 0.4746 | 0.031514 | 0.00451 | 1.29E-11 | FALSE | TRUE | 6.07E-08 | CMIP |
| rs2980879 | 8 | 126481475 | T | A | 0.375 | 0.029851 | 0.005059 | 1.08E-08 | TRUE | TRUE | 3.60E-07 | RP11-136O12.2 |
| rs601339 | 12 | 123174743 | G | A | 0.15 | 0.039007 | 0.005719 | 3.87E-11 | FALSE | TRUE | 2.10E-08 | RP11-324E6.6 |
| rs6810075 | 3 | 186548565 | C | T | 0.6333 | -0.066402 | 0.004788 | 1.00E-200 | FALSE | TRUE | 1.28E-31 | RP11-573D15.1 |
| rs731839 | 19 | 33899065 | A | G | 0.6724 | 0.036626 | 0.004838 | 2.20E-13 | FALSE | TRUE | 1.43E-11 | PEPD |
| rs7615090 | 3 | 186591003 | G | T | 0.8833 | -0.058149 | 0.008464 | 2.81E-11 | FALSE | TRUE | 1.48E-08 | TMEM207 |
| rs7955516 | 12 | 20498036 | C | A | 0.4417 | 0.026448 | 0.004594 | 2.43E-08 | FALSE | TRUE | 4.49E-07 | RP11-284H19.1 |
| rs7964945 | 12 | 124437668 | A | T | 0.8083 | 0.036875 | 0.006419 | 2.61E-08 | TRUE | TRUE | 5.56E-07 | CCDC92 |
