# Supplementary material for: Genetically predicted adiponectin causally reduces the risk of chronic kidney disease, a bilateral and multivariable mendelian randomization study
Source: Front Genet. 2022 Jul 26;13:920510. doi: 10.3389/fgene.2022.920510 (PMC9360570; doi:10.3389/fgene.2022.920510)
Supplement: Supplementary file 5 [file Table2.DOCX]

| **SNP** | **chr** | **pos** | **A1** | **A2** | **EAF** | **Beta** | **palindromic** | **ambiguous** | **SE** | **P value** | **Steiger**  **P value** |
| --- | --- | --- | --- | --- | --- | --- | --- | --- | --- | --- | --- |
| rs13333226 | 16 | 20365654 | G | A | 0.177 | -0.21 | FALSE | FALSE | 0.02 | 3.80E-26 | 5.52E-05 |
| rs2453533 | 15 | 45641225 | A | C | 0.291 | 0.11 | FALSE | FALSE | 0.015 | 5.40E-12 | 0.011365 |
| rs3812035 | 5 | 176817143 | T | G | 0.367 | 0.1 | FALSE | FALSE | 0.017 | 2.90E-09 | 0.731085 |
| rs7805747 | 7 | 151407801 | A | G | 0.296 | 0.14 | FALSE | FALSE | 0.019 | 2.10E-14 | 0.023205 |
