# Supplementary material for: Genetically predicted adiponectin causally reduces the risk of chronic kidney disease, a bilateral and multivariable mendelian randomization study
Source: Front Genet. 2022 Jul 26;13:920510. doi: 10.3389/fgene.2022.920510 (PMC9360570; doi:10.3389/fgene.2022.920510)
Supplement: Supplementary file 6 [file Table3.DOCX]

| **SNP** | **chr** | **pos** | **A1** | **A2** | **EAF** | **Beta** | **SE** | **P value** | **Steiger**  **P value** |
| --- | --- | --- | --- | --- | --- | --- | --- | --- | --- |
| rs1044261 | 10 | 1065710 | T | C | 0.084 | -0.011 | 0.0016 | 1.20E-11 | 6.59E-03 |
| rs10774021 | 12 | 349298 | T | C | 0.695 | -0.0063 | 0.00092 | 4.80E-12 | 3.39E-02 |
| rs10994856 | 10 | 52645248 | A | G | 0.186 | 0.0075 | 0.0011 | 1.20E-10 | 3.16E-02 |
| rs11613352 | 12 | 57792580 | T | C | 0.25 | 0.0057 | 0.001 | 4.70E-08 | 3.53E-02 |
| rs11655511 | 17 | 59287269 | T | C | 0.779 | -0.0083 | 0.0011 | 1.00E-13 | 9.40E-02 |
| rs11657044 | 17 | 59450105 | C | T | 0.82 | 0.011 | 0.0012 | 7.90E-22 | 7.15E-04 |
| rs12460876 | 19 | 33356891 | C | T | 0.423 | 0.0066 | 0.00092 | 1.90E-13 | 6.15E-03 |
| rs1260326 | 2 | 27730940 | C | T | 0.58 | -0.0068 | 0.00092 | 3.40E-14 | 9.61E-01 |
| rs13329952 | 16 | 20366507 | C | T | 0.217 | 0.016 | 0.0011 | 9.50E-43 | 8.35E-08 |
| rs1394125 | 15 | 76158983 | A | G | 0.35 | -0.0073 | 0.001 | 5.50E-14 | 1.24E-02 |
| rs1405809 | 7 | 156266712 | A | G | 0.235 | 0.0064 | 0.001 | 8.30E-10 | 2.87E-02 |
| rs163160 | 11 | 2789955 | G | A | 0.143 | -0.0067 | 0.0011 | 9.70E-09 | 1.11E-02 |
| rs17216707 | 20 | 52732362 | C | T | 0.212 | 0.0084 | 0.0011 | 6.00E-13 | 1.44E-03 |
| rs17319721 | 4 | 77368847 | A | G | 0.42 | -0.011 | 0.00092 | 1.30E-37 | 2.85E-06 |
| rs1800615 | 1 | 15832281 | T | C | 0.231 | -0.0058 | 0.00092 | 1.90E-09 | 8.40E-02 |
| rs228611 | 4 | 103561709 | A | G | 0.447 | -0.0055 | 0.00092 | 4.70E-10 | 2.34E-02 |
| rs2467853 | 15 | 45698793 | G | T | 0.328 | -0.013 | 0.00092 | 1.00E-42 | 7.03E-08 |
| rs267734 | 1 | 150951477 | C | T | 0.208 | 0.0079 | 0.0011 | 4.00E-13 | 8.00E-03 |
| rs2861422 | 3 | 141724644 | T | C | 0.248 | 0.0074 | 0.001 | 9.10E-14 | 6.57E-03 |
| rs316009 | 6 | 160675764 | C | T | 0.903 | -0.013 | 0.0014 | 4.40E-19 | 3.09E-03 |
| rs3758086 | 8 | 23714992 | A | G | 0.446 | -0.0071 | 0.00092 | 1.70E-15 | 2.04E-01 |
| rs3820716 | 2 | 148680260 | A | G | 0.5 | -0.0052 | 0.00092 | 2.70E-09 | 3.83E-01 |
| rs3850625 | 1 | 201016296 | A | G | 0.097 | 0.008 | 0.0014 | 6.40E-09 | 2.13E-01 |
| rs4744712 | 9 | 71434707 | C | A | 0.617 | 0.0071 | 0.00092 | 4.30E-15 | 9.35E-03 |
| rs491567 | 15 | 53946593 | C | A | 0.208 | 0.0084 | 0.001 | 2.90E-15 | 2.08E-03 |
| rs6420094 | 5 | 176817636 | G | A | 0.367 | -0.0096 | 0.001 | 4.90E-22 | 5.90E-02 |
| rs6503507 | 17 | 37525274 | T | C | 0.27 | 0.0076 | 0.001 | 1.50E-14 | 1.87E-03 |
| rs6546838 | 2 | 73679280 | G | A | 0.252 | 0.0093 | 0.001 | 7.70E-20 | 4.18E-03 |
| rs6795744 | 3 | 13906850 | A | G | 0.133 | 0.0071 | 0.0012 | 9.60E-09 | 1.65E-02 |
| rs715 | 2 | 211543055 | C | T | 0.292 | -0.0096 | 0.001 | 2.30E-21 | 2.62E-03 |
| rs7805747 | 7 | 151407801 | A | G | 0.296 | -0.013 | 0.0011 | 8.00E-29 | 7.33E-05 |
| rs7956634 | 12 | 15321194 | C | T | 0.174 | 0.0068 | 0.0011 | 2.50E-09 | 2.54E-02 |
| rs807601 | 2 | 15793014 | T | G | 0.319 | 0.0064 | 0.00092 | 6.60E-12 | 2.06E-02 |
| rs894680 | 17 | 19440538 | A | G | 0.42 | -0.0065 | 0.00092 | 1.00E-11 | 1.30E-01 |
| rs913423 | 10 | 94845036 | A | G | 0.535 | -0.0052 | 0.00092 | 5.10E-09 | 1.82E-01 |
| rs9472135 | 6 | 43809802 | C | T | 0.266 | 0.008 | 0.001 | 3.30E-15 | 8.50E-02 |
| rs963837 | 11 | 30749090 | C | T | 0.456 | 0.0078 | 0.00092 | 5.70E-18 | 2.44E-02 |
