# Supplementary material for: Genetically predicted adiponectin causally reduces the risk of chronic kidney disease, a bilateral and multivariable mendelian randomization study
Source: Front Genet. 2022 Jul 26;13:920510. doi: 10.3389/fgene.2022.920510 (PMC9360570; doi:10.3389/fgene.2022.920510)
Supplement: Supplementary file 7 [file Table4.DOCX]

**Table 2.** MR estimates from each method of the causal effect of adiponectin on kidney function.

| **Traits** | **MR method** | **Cochran's Q statistic** | **Heterogeneity P value** | **MR-Egger intercept** | **Intercept P value** |
| --- | --- | --- | --- | --- | --- |
| **CKD** | MR-Egger | 6.27 | 0.617 | -0.0006 | 0.961 |
|  | IVW | 6.28 | 0.712 | - | - |
|  | Maximum likelihood method | 6.21 | 0.719 | - | - |
| **eGFR** | MR-Egger | 5.06 | 0.751 | 0.0013 | 0.089 |
|  | IVW | 8.81 | 0.455 | - | - |
|  | Maximum likelihood method | 8.77 | 0.459 | - | - |

MR: Mendelian randomization; IVW: Inverse variance weighted method.
